# Quantum criticality in an organic spin-liquid insulator *κ*-(BEDT-TTF)_2_Cu_2_(CN)_3_

**DOI:** 10.1038/ncomms13494

**Published:** 2016-11-14

**Authors:** Takayuki Isono, Taichi Terashima, Kazuya Miyagawa, Kazushi Kanoda, Shinya Uji

**Affiliations:** 1 National Institute for Materials Science, Tsukuba, Ibaraki 305-0003, Japan; 2 Department of Applied Physics, University of Tokyo, Bunkyo-ku, Tokyo 113-8656, Japan

## Abstract

A quantum spin-liquid state, an exotic state of matter, appears when strong quantum fluctuations enhanced by competing exchange interactions suppress a magnetically ordered state. Generally, when an ordered state is continuously suppressed to 0 K by an external parameter, a quantum phase transition occurs. It exhibits critical scaling behaviour, characterized only by a few basic properties such as dimensions and symmetry. Here we report the low-temperature magnetic torque measurements in an organic triangular-lattice antiferromagnet, *κ*-(BEDT-TTF)_2_Cu_2_(CN)_3_, where BEDT-TTF stands for bis(ethylenedithio)tetrathiafulvalene. It is found that the magnetic susceptibilities derived from the torque data exhibit a universal critical scaling, indicating the quantum critical point at zero magnetic field, and the critical exponents, *γ*=0.83(6) and *νz*=1.0(1). These exponents greatly constrain the theoretical models for the quantum spin liquid, and at present, there is no theory to explain the values, to the best of our knowledge.

## Abstract (web summary)

Quantum spin liquids emerge when quantum fluctuations suppress a magnetically ordered state. Here the authors measure magnetic torque in *κ*-(BEDT-TTF)_2_Cu_2_(CN)_3_, showing universal critical scaling in the magnetic susceptibilities, with critical exponents incompatible with known models for quantum spin liquids.

Strongly interacting spin systems generally form a magnetic order at sufficiently low temperatures. A remarkable exception is a quantum spin liquid (QSL), which is characterized by the presence of no spontaneous symmetry breaking, and unique magnetic excitations, called spinons^1^,^2^,^3^. Such a liquid state is in fact found in several geometrically frustrated spin systems, where strong quantum fluctuations enhanced by the spin frustration hinders the development of the conventional magnetic order; the magnetic transition temperature is reduced down to 0 K (ref. 4). In general, when an ordered state is continuously suppressed to 0 K by tuning an external parameter, for example, pressure, magnetic field or chemical composition, a quantum phase transition occurs. At the transition point, a quantum critical point (QCP), the system exhibits characteristic scale invariance in both space and time, and consequently, thermodynamic and dynamic quantities obey a universal scaling relation^4^,^5^,^6^,^7^. The critical exponents obtained by this scaling do not depend on microscopic details of the system, but only on a few fundamental properties such as spatial dimensions, and order-parameter symmetry. Therefore, an experimental determination of the exponents in the QSL systems provides the primary basis for understanding the QSL state.

There are several candidate materials for QSL states in geometrically frustrated antiferromagnetic (AF) insulators, such as triangular-lattice^8^,^9^,^10^ and kagome-lattice systems^11^,^12^. Among them, the organic triangular-lattice antiferromagnet, *κ*-(BEDT-TTF)_2_Cu_2_(CN)_3_, where BEDT-TTF stands for bis(ethylenedithio)tetrathiafulvalene, is a prime example for studying the QSL state, because of (1) much less impurities than the others^13^ and (2) the possible presence of the quantum criticality. In *κ*-(BEDT-TTF)_2_Cu_2_(CN)_3_, an *S*=1/2 spin is located on a 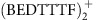 molecular dimer [dotted ellipsoid in Fig. 1a], forming a triangular lattice, and interacts with the nearest-neighbor spins by AF exchange coupling *J*. Albeit the large coupling constant *J*/*k*_B_∼250 K, no magnetic long-range order happens even at a very low temperature, *T*∼30 mK, which is four orders of magnitude lower than *J*/*k*_B_ (ref. 8). This result suggests that strong quantum fluctuations enhanced by the geometrical frustration suppress AF ordering, and consequently lead to a QSL state. In fact, the presence of the linear-in-temperature term in the specific heat shows that low-energy spin excitations in this state are gapless^14^. By contrast, the presence of a tiny gap of about 0.5 K is indicated by a thermally activated behaviour in the thermal conductivity^15^. Recently, the muon spin rotation (*μ*SR) study shows that there exists a QCP at a small field of ∼10 mT, separating a QSL state with a small gap of 3.5 mK and an AF state with a strongly suppressed magnetic moment^16^. To explain these exotic features, various theoretical models have been proposed^17^,^18^,^19^,^20^,^21^, but the interpretations are highly controversial.

Here we report the results of torque magnetometry on *κ*-(BEDT-TTF)_2_Cu_2_(CN)_3_, which unveil the universal critical scaling of the magnetic susceptibility. The scaling analyses show the QCP at zero magnetic field, consistent with the previous *μ*SR study, and the critical exponents, *γ*=0.83(6) and *νz*=1.0(1). To our best knowledge, there is no theory to explain these values, at present.

## Results

### Field-angle dependence of the magnetic torque

**Figure 1:**
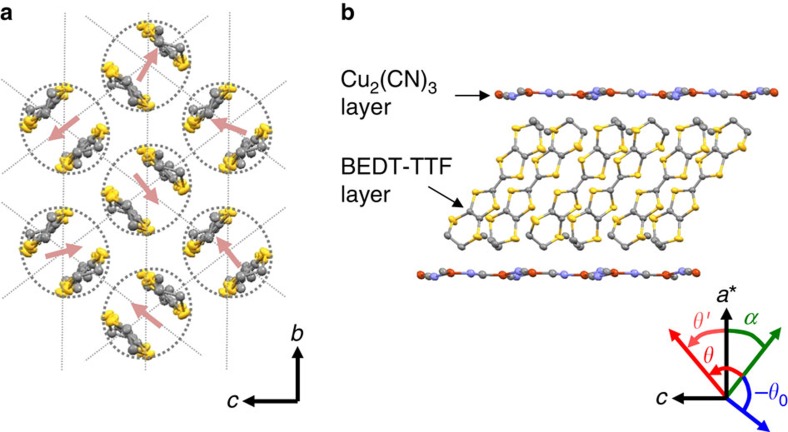
Crystal structure of *κ*-(BEDT-TTF)_2_Cu_2_(CN)_3_. (**a**) Molecular arrangement in a two-dimensional magnetic layer. The dotted ellipsoid and arrow stand for a 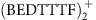 molecular dimer, which constitutes a triangular lattice, and an *S*=1/2 spin on the dimer, respectively. (**b**) Two-dimensional layered structure viewed in the crystallographic a*−*c* plane. The symbols *θ* and *θ*_0_ define the field angle in the torque measurements, and *θ*′ and *α* define the field angle in the calculation of the magnetic susceptibility (see the ‘Methods' section). The yellow, grey, blue and red ellipsoids represent the sulfur, carbon, nitrogen and copper atoms, respectively. The hydrogen atoms are omitted for clarity.

**Figure 2:**
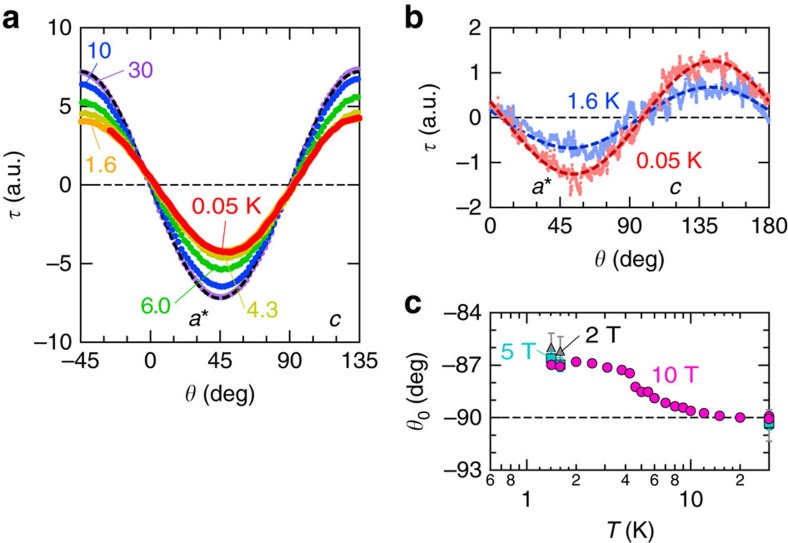
Magnetic torque data. (**a**) The field-angle dependence of the magnetic torque *τ*(*θ*) at *μ*_0_*H*=10 T. A magnetic field is applied parallel to the a*−*c* plane. The dashed curve at *T*=30 K shows *τ*(*θ*)∝Δ*χ*sin2(*θ*−*θ*_0_), where Δ*χ* and *θ*_0_ denote anisotropic susceptibility and a phase factor, respectively. (**b**) *τ*(*θ*) at *μ*_0_*H*=1 T. The dashed curve shows *τ*(*θ*)∝Δ*χ*sin2(*θ*−*θ*_0_). (**c**) The temperature variation in *θ*_0_ for the field rotation within the a*−*c* plane. The error bars represent the s.d. in the sinusoidal fits.

The main purpose of our study is to perform the critical scaling analysis on the static magnetic susceptibility. First, we have measured the field-angle dependence of the magnetic torque, *τ*(*θ*), to precisely obtain the magnetic susceptibility down to millikelvin temperatures. Figure 2a displays *τ*(*θ*) for *μ*_0_*H*=10 T parallel to the crystallographic a*−*c* plane (Fig. 1b). At *T*=30 K, the torque shows sinusoidal dependence, which is well described by a typical paramagnetic curve, *τ*(*θ*)∝Δ*χ*sin2(*θ*−*θ*_0_). Here, Δ*χ* and *θ*_0_ represent anisotropic susceptibility and a phase factor, respectively. The orientations of the principal axes determined by the angle at which *τ*=0 agree with those expected from the anisotropy of the *g* factor at room temperature^13^. The above results clearly show that the torque signal arises from the paramagnetic spins on the triangular lattice composed of the BEDT-TTF dimers. As temperature is lowered, Δ*χ* gradually decreases, consistent with the previous results^8^, and becomes temperature independent below *T*=1.6 K. At *μ*_0_*H*=1 T, on the other hand, an increase of Δ*χ* is observed with decreasing temperature below *T*=1.6 K (Fig. 2b). The *θ*_0_ value has a tendency to increase below *T*∼10 K, and subsequently reaches a constant value, approximately −87°, below *T*∼3 K, nearly regardless of the strength of a magnetic field, as shown in Fig. 2c. The change of *θ*_0_, the change of the principal axis of the magnetization, could be attributed to some structural origin.

### Magnetic susceptibility estimated by the torque

**Figure 3:**
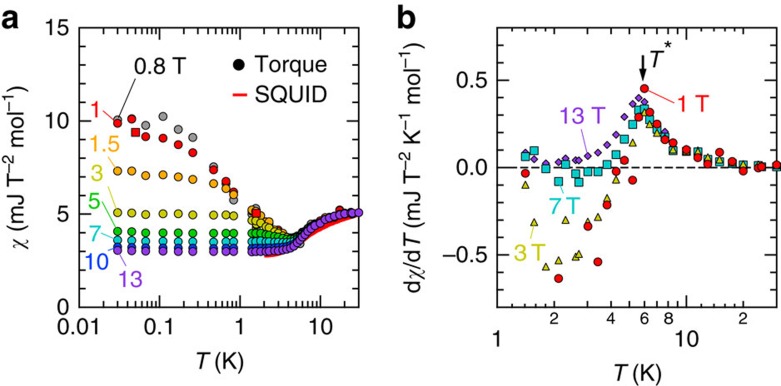
Magnetic susceptibility estimated by the torque data. (**a**) Static magnetic susceptibility as a function of temperature. The previously reported data by the SQUID magnetometry is also shown. (**b**) The temperature variation in the derivative of the magnetic susceptibility with respect to temperature. The arrow indicates a peak at a characteristic temperature T*=6 K.

From the paramagnetic-like *τ*(*θ*) curves shown above, we can estimate the static magnetic susceptibility *χ*, defined by the magnetization divided by a magnetic field *M*/*H*, based on the methodology reported in ref. 22 (see the ‘Methods' section for the details of the estimation). As shown in Fig. 3a, *χ* in low magnetic fields monotonically increases with decreasing temperature below *T*∼4 K. The increasing trend is suppressed by applying a magnetic field, and consequently *χ* becomes nearly independent of temperature in high magnetic fields. These *χ*(*T*) curves are significantly different from those in the QSL states of the other organic triangular-lattice magnets, where the Pauli-paramagnetic-like (field- and temperature-independent) behaviour is observed^10^,^22^. The temperature derivative of the susceptibility d*χ*/d*T* shows a clear peak at T*∼6 K, only below which the d*χ*/d*T* curves depend on a magnetic field, as shown in Fig. 3b. Therefore, the characteristic field dependence of *χ*(*T*) is attributed to the intrinsic effect, but not to impurity spins; the impurity effect should be observed even above T*. The characteristic temperature T* depends little on a magnetic field. Anomalies of physical quantities at T* have also been detected in the several experiments^14^,^15^,^23^. Possible scenarios, such as pairing instability of fermionic spinons^24^, modulation of spinon–phonon interactions^23^ and a characteristic temperature of a *Z*_2_ spin liquid^20^, have been proposed to explain these anomalies. However, no theory provides satisfactory explanations.

**Figure 4:**
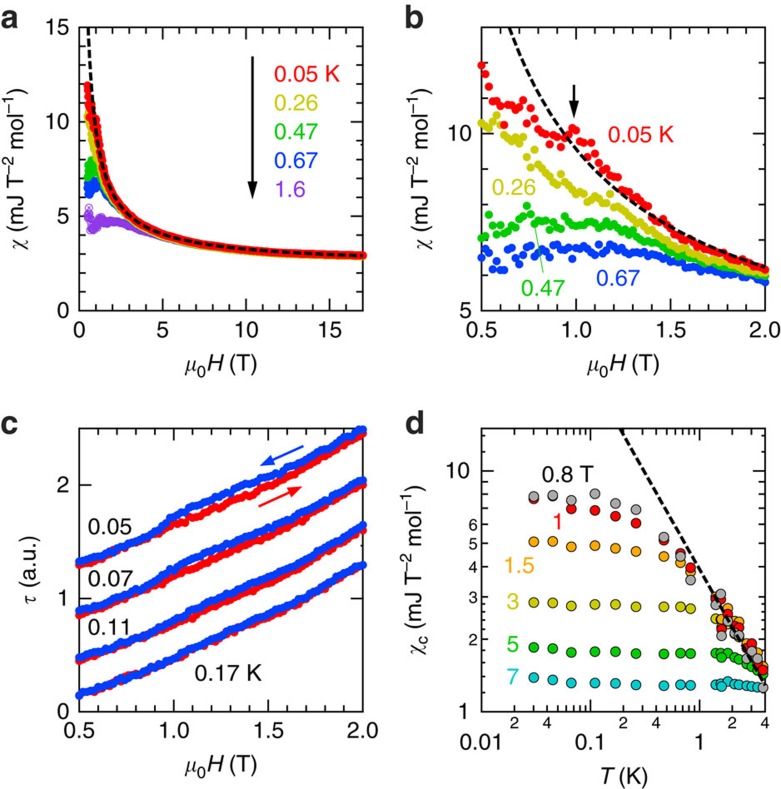
Power-law behaviours of the magnetic susceptibility. (**a**) Magnetic susceptibility as a function of a magnetic field *χ*(*H*). The dashed curve shows a power-law expression, *χ*(*H*)=*AH*^−*p*^+*χ*_0_ with a coefficient *A*, an exponent *P*=0.83, and constant susceptibility *χ*_0_=2.23 mJ T^−2^ mol^−1^. (**b**) The enlarged figure for a low-field region of **a**. The arrow denotes a kink at about *μ*_0_*H*=1 T. (**c**) The field dependence of the torque around *μ*_0_*H*=1 T. (**d**) The temperature dependence of the power-law component of the susceptibility, *χ*_c_=*χ*−*χ*_0_. The dashed line represents *χ*_c_(*T*)∝*T*^−*γ*^ with an exponent *γ*=0.8.

Next, let us focus on the magnetic-field dependence of the susceptibility *χ*(*H*) shown in Fig. 4a. A striking feature of *χ*(*H*) is a divergent behaviour as *H*→0 for *T*=0.05 K. Above 1 T, the field dependence can be well fitted with an expression (dashed curve), *χ*(*H*)=*χ*_c_(*H*)+*χ*_0_, where *χ*_c_(*H*)=*AH*^−*p*^ with a coefficient *A* and an exponent *p*=0.83, and constant susceptibility *χ*_0_=2.23 mJT^−2^ mol^−1^. Below 1 T (Fig. 4b), however, we see a deviation from the power-law expression (dashed curve). The power-law behaviour is suppressed with increasing temperature. Around *μ*_0_*H*=1 T, small hysteresis of the torque is observed at *T*=0.07 K and below, as shown in Fig. 4c, suggesting the presence of a first-order-like transition. In Fig. 4d, we note that *χ*_c_(*T*) also follows a power law, *χ*_c_(*T*)∝*T*^−*γ*^ with an exponent *γ*∼0.8, in a relatively high-temperature region near T*=6 K.

### Scaling analysis

**Figure 5:**
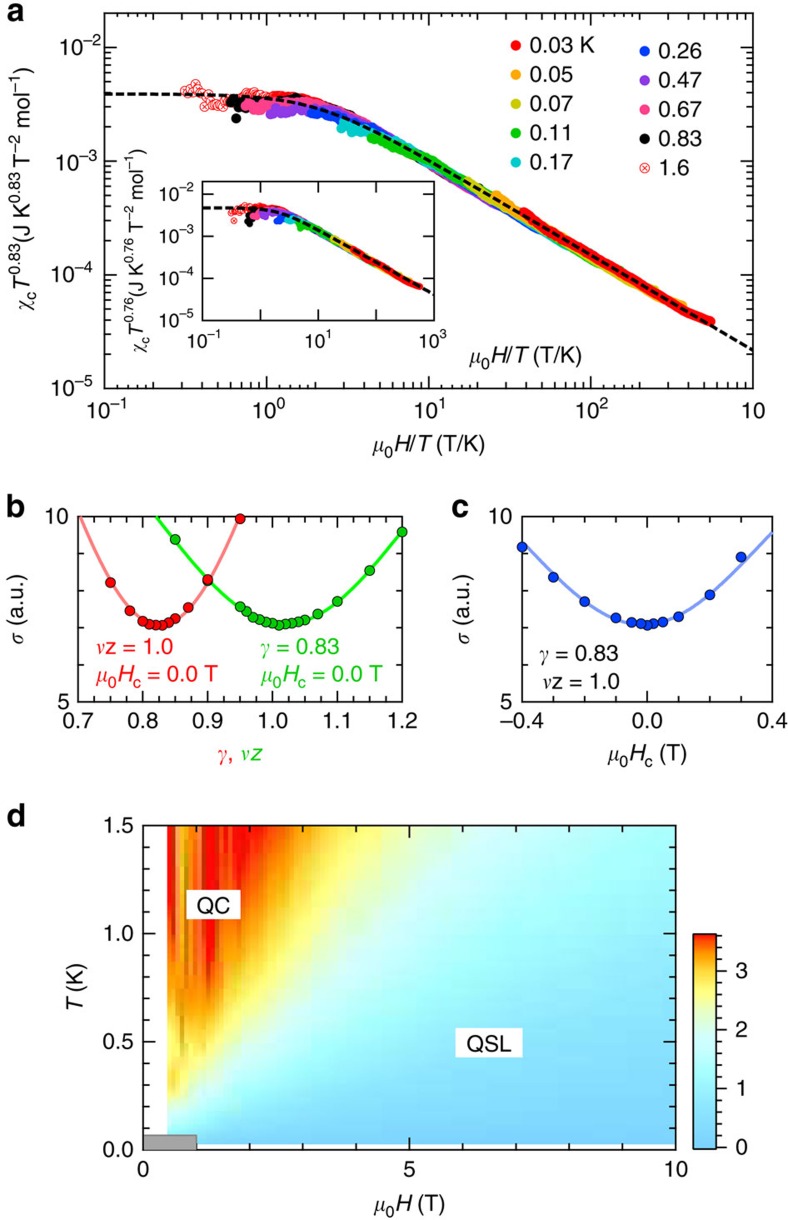
Critical scaling and quantum phase diagram. (**a**) Scaling plot of the power-law component of the susceptibility multiplied by the power of temperature *χ*_c_*T*^*γ*^ against a magnetic field divided by temperature *H*/*T*. The data for both *T*≤0.07 K and *μ*_0_*H*≤1.2 T are excluded from the figure for clarity. The dashed curve shows a scaling function, *f*(*x*)=*a*(*x*^2^+*b*^2^)^−*γ*/2^, where *a* and *b* represent certain constants, and *x*=(*H*−*H*_c_)/*T*^1/*νz*^. Inset: the scaling plot of the data for the a*−*b* field rotation in the different sample. The s.d., *σ*, of the scaling for various values of (**b**) *γ* and *νz*, and (**c**) *H*_c_, when the other two parameters are fixed. (**d**) Contour plot of *χ*_c_*T*^0.83^ in the *T*−*H* plane. The red and blue areas represent the quantum critical (QC) and quantum spin-liquid (QSL) regimes, respectively, which are bounded by the crossover region (yellow area). The scaling is broken in a low temperature and field region (shaded area).

We further examine the nature of the power-law behaviour by a scaling analysis. Figure 5a shows the scaling plot, *χ*_c_*T*^*γ*^ versus *H*/*T*. The data points fall on a universal curve over more than three decades in the *x* axis, and two decades in the *y* axis for *γ*=0.83. Almost the same scaling, *γ*=0.76, can be achieved for the *a*−b* field rotation in the different samples, as shown in the inset of Fig. 5a. Here, the data for *T*≤0.07 K and *μ*_0_*H*≤1.2 T, deviating from the universal curve, are omitted from the figure for clarity. The *H*/*T* scaling of thermodynamic and dynamic quantities induced by intrinsic quantum criticality^5^, and disorder^25^ has been reported for heavy-fermion systems near a QCP. The disorder-induced scaling will appear when there exists local distribution of a characteristic-energy scale in the system, and a distribution function has a finite weight near the zero energy^25^,^26^. For *κ*-(BEDT-TTF)_2_Cu_2_(CN)_3_, the distribution of the characteristic-energy scale *J* could be caused by charge imbalance between the two BEDT-TTF^0.5+^ molecules in a dimer. According to infrared vibrational spectroscopy, however, the charge imbalance is no more than ±0.005*e* (ref. 27), which must be too small to induce wide distribution of *J* down to 0 K. The disorder-induced scaling could also be inconsistent with the fact that the susceptibility is scaled with the almost same exponents in two different samples; the exponents are non-universal in the disorder scenario. Thus, the quantum criticality will be the dominant reason for the above *H*/*T* scaling.

Assuming that a critical behaviour is governed by a correlation length 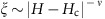 and correlation time 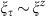, a critical contribution to the magnetic susceptibility is expected as *χ*_c_=*χ*−*χ*_0_=*T*^−*γ*^*f*[(*H*−*H*_c_)/*T*^1/*νz*^] (ref. 28). Here, *χ*_0_, *γ*, *ν*, *z* and *H*_c_ represent a regular component of *χ*, a critical exponent of *χ*_c_, a correlation-length exponent, a dynamic exponent and a critical field, respectively. As described above, *χ*_0_ is a temperature- and field-independent term. This is reasonable because such Pauli-paramagnetic-like susceptibility has been observed in another organic QSL material, EtMe_3_Sb[Pd(dmit)_2_]_2_, which appears to be located far from the QCP^22^. Our scaling form, *χ*_c_*T*^0.83^=*f*(*H*/*T*), clearly shows that (1) the presence of the QCP close to the zero field, and (2) the critical exponents, *γ*=0.83 and *νz*=1. The scaling behaviour in Fig. 5a is understood as the two distinct regimes bounded by a crossover region, 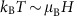; the quantum critical (QC) regime characterized by *χ*_c_*T*^0.83^=const in the low *H*/*T* region, and the QSL regime where the data collapse on the (*H*/*T*)^−0.83^ line in the high *H*/*T* region and hence *χ*_c_∝*H*^−0.83^. Note that the exponent in each region well agrees with the power of *χ*_c_(*H*) and *χ*_c_(*T*) (see Fig. 4a,d). To estimate a statistical error of the critical exponents and the critical field, we assume the scaling function, *f*(*x*)=*a*(*x*^2^+*b*^2^)^−*γ*/2^ (dashed curve in Fig. 5a), where *a* and *b* represent constants, and *x*=(*H*−*H*_c_)/*T*^1/*νz*^. This functional form satisfies the scaling behaviour in low- and high-*H*/*T* regions. The best collapse of the data onto this function is obtained for *γ*=0.83(6), *νz*=1.0(1) and *H*_c_=0.0(2) T. These values give the smallest standard deviation, as shown in Fig. 5b,c. Similarly, we obtain *γ*=0.76(9), *νz*=1.0(2) and *H*_c_=0.0(3) T for another sample (the inset of Fig. 5a). The obtained *νz* value agrees with the *μ*SR result, *νz*=0.94(1), within the error.

## Discussion

From the above results, we can make a contour plot of *χ*_c_*T*^*γ*^ (Fig. 5d), giving the *T*−*H* phase diagram. One finds the QC regime above the zero-field QCP, and the growth of the QSL regime by applying a magnetic field. Within the error, the zero-field QCP is consistent with the *μ*SR result, the QCP at the small field about 10 mT (ref. 16). In the low temperature and field region (shaded area), an ordered phase is present, where the scaling is broken. The ordered phase does not seem a simple AF state with a spin-flop transition, because of the sin2*θ* behaviour of *τ*(*θ*). By contrast, the *μ*SR study suggests a weak AF order in much wider field region^16^, where the magnetic torque and heat capacity do not detect any AF transition. The disagreement with the *μ*SR study could arise from the different observation time; the *μ*SR can detect AF correlation in a very fast timescale (nano- to microseconds), whereas the magnetic torque and heat capacity have much longer observation time. The AF order may be strongly fluctuated, which makes it quite difficult to detect a sign of the AF order by the torque and heat capacity.

In contrast to *κ*-(BEDT-TTF)_2_Cu_2_(CN)_3_, no sign of the quantum criticality has been observed in the other organic QSL systems, EtMe_3_Sb[Pd(dmit)_2_]_2_ and *κ*-H_3_(Cat-EDT-TTF)_2_, which will be located far from the QCP at the zero field. In these systems, the values of the Pauli-like *T*- and *H*-independent susceptibility *χ*_0_ are 4 mJ T^−2^ mol^−1^ for EtMe_3_Sb[Pd(dmit)_2_]_2_, and 12 mJ T^−2^ mol^−1^ for *κ*-H_3_(Cat-EDT-TTF)_2_. It is approximately inversely proportional to the AF exchange coupling constant, 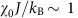 J K T^−2^ mol^−1^ (ref. 10), suggesting that *χ*_0_ is governed by the spinon density of states. If this relation also holds for *κ*-(BEDT-TTF)_2_Cu_2_(CN)_3_, the susceptibility is to be ∼4 mJ T^−2^ mol^−1^ from 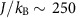 K. The non-critical part *χ*_0_=2.23 mJ T^−2^ mol^−1^ obtained from the scaling analysis suggests that about a half of the total *χ* contributes to the critical behaviour.

The critical exponents obtained here are different from those in the other candidates for the QSL state^7^,^29^. Unexpectedly, a typical heavy-fermion system, CeCu_5.9_Au_0.1_, has similar values, *γ*=0.75–0.80 and *νz*=1, which are determined from *H*/*T* scaling of *χ*, and *E*/*T* scaling of the dynamic susceptibility^5^,^28^. Further studies will be required to clarify if this heavy-fermion system and the present organic AF insulator share the same universality class. A possible scenario for the quantum criticality in heavy-fermion systems is a Kondo-breakdown transition to an exotic Fermi-liquid state, where the localized *f*-electrons form a spin liquid^30^. The critical exponents determined in the present study greatly constrain the theoretical models for the QSL state in the organic triangular-lattice systems, and now there is no theory to explain the experimental values, to our best knowledge.

## Methods

### Sample preparation and torque magnetometry

Single-crystalline samples were prepared by the electrochemical oxidation of the BEDT-TTF molecules. A selected single crystal was mounted on a micro-cantilever to measure the magnetic torque. Thanks to the high sensitivity of this method, we could detect the considerably small paramagnetic torque on the single crystal with typical mass of about 50 μg. All the experiments were made using a 20 T superconducting magnet with a dilution refrigerator, and a 17 T superconducting magnet with a ^4^He variable temperature insert at Tsukuba Magnet Laboratory, NIMS. A magnetic field is applied parallel to the crystallographic a*−*b* and a*−*c* planes shown in Fig. 1b. The experiments for the two field directions give the qualitatively same results, and thus the results for the field rotation within the a*−*c* plane are mainly discussed in the text.

### Estimation of the magnetic susceptibility from the torque

The fundamental methodology for the estimation has already been provided in ref. 22. The main assumption is that the magnetic susceptibility is proportional to the square of the *g* factor, 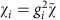, similar to the case for conventional paramagnets. Here, *χ*_*i*_, *g*_*i*_ and 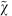 denote the static magnetic susceptibility along the principal axis, the *g* factor along its axis and the uniform susceptibility, respectively. Then, the magnetic torque for the field rotation within the a*−*c* plain is expressed by:


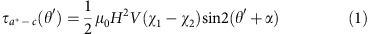



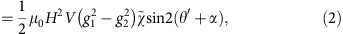


and for the a*−*b* rotation we obtain:


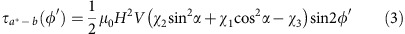



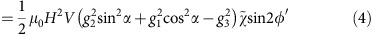


with the sample volume *V*, and *α*=38°, which is the angle between the molecular long axis and the a* axis, shown in Fig. 1b. This expression indeed succeeded to reveal *χ* in the QSL states of the organic triangular-lattice magnets, EtMe_3_Sb[Pd(dmit)_2_]_2_ and *κ*-H_3_(Cat-EDT-TTF)_2_ (refs 10, 22). For the present system, the electron-spin-resonance study reported *g*_1_=2.008, *g*_2_=2.005 and *g*_3_=2.003 (ref. 13). These values are nearly temperature independent down to *T*∼2 K, at which the small change of *θ*_0_ already occurs (see Fig. 2c). Thus, we naturally assume that the *g* factor is little affected by temperature changes below 2 K as well. Estimated 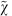 is normalized to *χ* at *T*=30 K measured by a SQUID magnetometer^8^, because the torque measurement for a tiny crystal includes some ambiguity in the absolute value. The normalization is ensured by the good agreement between *χ*(*T*) estimated here and measured by the SQUID in the wide temperature range.

### Data availability

The data that support the findings of this study are available from the corresponding author upon reasonable request.

## Additional information

**How to cite this article:** Isono, T. *et al.* Quantum criticality in an organic spin-liquid insulator *κ*-(BEDT-TTF)_2_Cu_2_(CN)_3_. *Nat. Commun.* **7,** 13494 doi: 10.1038/ncomms13494 (2016).

**Publisher's note:** Springer Nature remains neutral with regard to jurisdictional claims in published maps and institutional affiliations.

## References

[b1] Anderson P. W. Resonating valence bonds: a new kind of insulator. Mat. Res. Bull. 8, 153–160 (1973).

[b2] Balents L. Spin liquids in frustrated magnets. Nature 464, 199–208 (2010). 20220838 10.1038/nature08917

[b3] Normand B. Frontiers in frustrated magnetism. Contemp. Phys. 50, 533–552 (2009).

[b4] Sachdev S. Quantum Phase Transitions Second Edition Cambridge Univ. Press (2011).

[b5] Schröder A. *et al.* Onset of antiferromagnetism in heavy-fermion metals. Nature 407, 351–355 (2000). 11014185 10.1038/35030039

[b6] Matsumoto Y. *et al.* Quantum criticality without tuning in the mixed valence compound *β*-YbAlB_4_. Science 331, 316–319 (2011). 21252341 10.1126/science.1197531

[b7] Tokiwa Y., Ishikawa J. J., Nakatsuji S. & Gegenwart P. Quantum criticality in a metallic spin liquid. Nat. Mater 13, 356–359 (2014). 24651428 10.1038/nmat3900

[b8] Shimizu Y., Miyagawa K., Kanoda K., Maesato M. & Saito G. Spin liquid state in an organic Mott insulator with a triangular lattice. Phys. Rev. Lett. 91, 107001 (2003). 14525498 10.1103/PhysRevLett.91.107001

[b9] Itou T., Oyamada A., Maegawa S., Tamura M. & Kato R. Quantum spin liquid in the spin-1/2 triangular antiferromagnet EtMe_3_Sb[Pd(dmit)_2_]_2_. Phys. Rev. B 77, 104413 (2008).

[b10] Isono T. *et al.* Gapless quantum spin liquid in an organic spin-1/2 triangular lattice *κ*-H_3_(Cat-EDT-TTF)_2_. Phys. Rev. Lett. 112, 177201 (2014). 24836269 10.1103/PhysRevLett.112.177201

[b11] Han T. H. *et al.* Fractionalized excitations in the spin-liquid state of a kagome-lattice antiferromagnet. Nature 492, 406–410 (2012). 23257883 10.1038/nature11659

[b12] Clark L. *et al.* Gapless spin liquid ground state in the S=1/2 vanadium oxyfluoride kagome antiferromagnet [NH_4_]_2_[C_7_H_14_N][V_7_O_6_F_18_]. Phys. Rev. Lett. 110, 207208 (2013). 25167449 10.1103/PhysRevLett.110.207208

[b13] Komatsu T., Matsukawa N., Inoue T. & Saito G. Realization of superconductivity at ambient pressure by band-filling control in *κ*-(BEDT-TTF)_2_Cu_2_(CN)_3_. J. Phys. Soc. Jpn. 65, 1340–1354 (1996).

[b14] Yamashita S. *et al.* Thermodynamic properties of a spin-1/2 spin-liquid state in a *κ*-type organic salt. Nat. Phys 4, 459–462 (2008).

[b15] Yamashita M. *et al.* Thermal-transport measurements in a quantum spin-liquid state of the frustrated triangular magnet *κ*-(BEDT-TTF)_2_Cu_2_(CN)_3_. Nat. Phys 5, 44–47 (2009).

[b16] Pratt F. L. *et al.* Magnetic and non-magnetic phases of a quantum spin liquid. Nature 471, 612–616 (2011). 21455176 10.1038/nature09910

[b17] Hayashi Y. & Ogata M. Possibility of gapless spin liquid state by one-dimensionalization. J. Phys. Soc. Jpn. 76, 053705 (2007).

[b18] Motrunich O. I. Variational study of triangular lattice spin-1/2 model with ring exchanges and spin liquid state in *κ*-(ET)_2_Cu_2_(CN)_3_. Phys. Rev. B 72, 045105 (2005).

[b19] Lee S. S. & Lee P. A. U(1) gauge theory of the Hubbard model: spin liquid states and possible application to *κ*-(BEDT-TTF)_2_Cu_2_(CN)_3_. Phys. Rev. Lett. 95, 036403 (2005). 16090761 10.1103/PhysRevLett.95.036403

[b20] Qi Y., Xu C. & Sachdev S. Dynamics and transport of the Z_2_ spin liquid: application to *κ*-(ET)_2_Cu_2_(CN)_3_. Phys. Rev. Lett. 102, 176401 (2009). 19518801 10.1103/PhysRevLett.102.176401

[b21] Watanabe K., Kawamura H., Nakano H. & Sakai T. Quantum spin-liquid behavior in the spin-1/2 random Heisenberg antiferromagnet on the triangular lattice. J. Phys. Soc. Jpn. 83, 034714 (2014).

[b22] Watanabe D. *et al.* Novel Pauli-paramagnetic quantum phase in a Mott insulator. Nat. Commun. 3, 1090 (2012). 23011144 10.1038/ncomms2082

[b23] Poirier M., de Lafontaine M., Miyagawa K., Kanoda K. & Shimizu Y. Ultrasonic investigation of the transition at 6 K in the spin-liquid candidate *κ*-(BEDT-TTF)_2_Cu_2_(CN)_3_. Phys. Rev. B 89, 045138 (2014).

[b24] Lee S. S., Lee P. A. & Senthil T. Amperean pairing instability in the U(1) spin liquid state with Fermi surface and application to *κ*-(BEDT-TTF)_2_Cu_2_(CN)_3_. Phys. Rev. Lett. 98, 067006 (2007). 17358978 10.1103/PhysRevLett.98.067006

[b25] Tabata Y. *et al.* *H*/*T* scaling in disordered non-Fermi liquid materials Ce(Ru_1−*x*_Rh_*x*_)Si_2_ for *x*=0.5 and 0.6: Quantum Griffiths nature. Phys. Rev. B 70, 144415 (2004).

[b26] Castro Neto A. H. & Jones B. A. Non-Fermi-liquid behavior in U and Ce alloys: criticality, disorder, dissipation, and Griffiths-McCoy singularities. Phys. Rev. B 62, 14975–15011 (2000).

[b27] Sedlmeier K. *et al.* Absence of charge order in the dimerized *κ*-phase BEDT-TTF salts. Phys. Rev. B 86, 245103 (2012).

[b28] Löhneysen H. v., Rosch A., Vojta M. & Wölfle P. Fermi-liquid instabilities at magnetic quantum phase transitions. Rev. Mod. Phys. 79, 1015–1075 (2007).

[b29] Helton J. S. *et al.* Dynamic scaling in the susceptibility of the spin-1/2 kagome lattice antiferromagnet herbertsmithite. Phys. Rev. Lett. 104, 147201 (2010). 20481955 10.1103/PhysRevLett.104.147201

[b30] Senthil T., Vojta M. & Sachdev S. Weak magnetism and non-Fermi liquids near heavy-fermion critical points. Phys. Rev. B 69, 035111 (2004).

